# ReCiter: An open source, identity-driven, authorship prediction algorithm optimized for academic institutions

**DOI:** 10.1371/journal.pone.0244641

**Published:** 2021-04-01

**Authors:** Paul J. Albert, Sarbajit Dutta, Jie Lin, Zimeng Zhu, Michael Bales, Stephen B. Johnson, Mohammad Mansour, Drew Wright, Terrie R. Wheeler, Curtis L. Cole

**Affiliations:** 1 Samuel J. Wood Library and Information Technologies & Services, Weill Cornell Medicine, New York, New York, United States of America; 2 Information Technologies & Services, Weill Cornell Medicine, New York, New York, United States of America; 3 Department of Radiology, Weill Cornell Medicine, New York, New York, United States of America; 4 Connective Media Program, Cornell University, Cornell Tech, New York, New York, United States of America; 5 Samuel J. Wood Library, Weill Cornell Medicine, New York, New York, United States of America; 6 New York University Langone Health, New York, New York, United States of America; Indiana University Bloomington, UNITED STATES

## Abstract

Academic institutions need to maintain publication lists for thousands of faculty and other scholars. Automated tools are essential to minimize the need for direct feedback from the scholars themselves who are practically unable to commit necessary effort to keep the data accurate. In relying exclusively on clustering techniques, author disambiguation applications fail to satisfy key use cases of academic institutions. Algorithms can perfectly group together a set of publications authored by a common individual, but, for them to be useful to an academic institution, they need to programmatically and recurrently map articles to thousands of scholars of interest *en masse*. Consistent with a savvy librarian’s approach for generating a scholar’s list of publications, identity-driven authorship prediction is the process of using information about a scholar to quantify the likelihood that person wrote certain articles. ReCiter is an application that attempts to do exactly that. ReCiter uses institutionally-maintained identity data such as name of department and year of terminal degree to predict which articles a given scholar has authored. To compute the overall score for a given candidate article from PubMed (and, optionally, Scopus), ReCiter uses: up to 12 types of commonly available, identity data; whether other members of a cluster have been accepted or rejected by a user; and the average score of a cluster. In addition, ReCiter provides scoring and qualitative evidence supporting why particular articles are suggested. This context and confidence scoring allows curators to more accurately provide feedback on behalf of scholars. To help users to more efficiently curate publication lists, we used a support vector machine analysis to optimize the scoring of the ReCiter algorithm. In our analysis of a diverse test group of 500 scholars at an academic private medical center, ReCiter correctly predicted 98% of their publications in PubMed.

## 1. Introduction

Author name disambiguation is the process of inferring, often using clustering techniques, whether the same author who wrote one paper wrote another paper. Many software initiatives have been engineered for the purpose of disambiguating author names in Medline data, and a subset of these have code that is publicly available for use. Author disambiguation software can be divided into those that perform unsupervised and supervised approaches. Techniques for unsupervised approaches generally involve clustering-based algorithms. One paper [1] discusses a K-way spectral clustering approach using features such as co-author names, paper titles, and publication venue titles. Another [2] describes an agglomerative clustering algorithm approach with pairwise similarity to disambiguate author names specifically for PubMed using features such as title, affiliation, journal, co-authors, etc. Techniques for supervised learning include naive Bayes probability model and support vector machines (SVM) [3]. A more recent author disambiguation approach uses deep learning [4]. A hybrid approach [5] using both supervised and unsupervised approach was also developed using a co-authorship graph and subset selection. Generally speaking, significant progress has been made, with several systems having a claimed accuracy in excess of 97% [6, 7].

While there has been marked success in clustering accuracy (bearing in mind that any quoted accuracy figure depends on context), an entirely different question may be far more pressing to many academic institutions: which papers did a given person write? Academic institutions are called upon to regularly update lists of publications for thousands of their scholars including papers authored at prior institutions or, in the case of alumni, at succeeding institutions. Some scholars are established principal investigators while others are second-year graduate students and do not even have a single publication to their name. Often times, institutions must maintain such lists with limited feedback from the scholars themselves; in other cases, feedback is provided, but it is not timely. Authorship prediction is non-trivial. For example, Weill Cornell Medicine has a father and son duo who share identical first and last names, and both publish in the field of plastic and reconstructive surgery. While an author disambiguation algorithm could output two perfectly disambiguated clusters for these individuals, these articles also need to be programmatically tied back to them.

**Fig 1:**
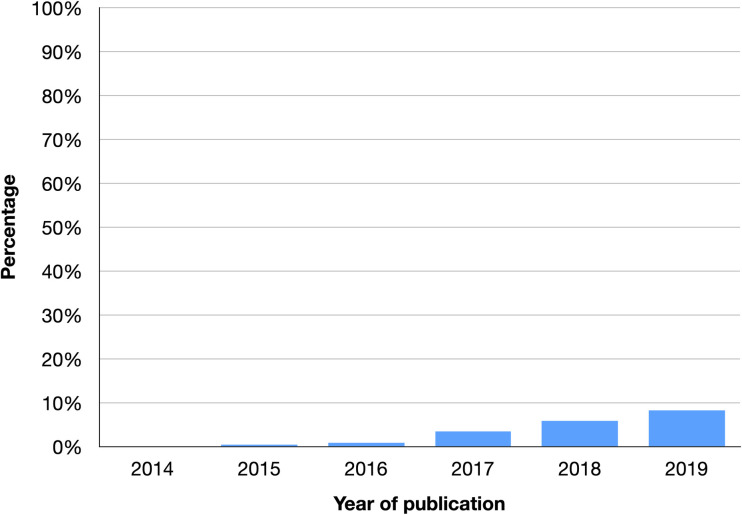
ORCID coverage in PubMed. Fewer than 6% of Weill Cornell papers in PubMed have an ORCID asserted. Of the subset of papers published in 2019, only 22% or 1 in 5 authors has an ORCID represented in PubMed.

**Fig 2:**
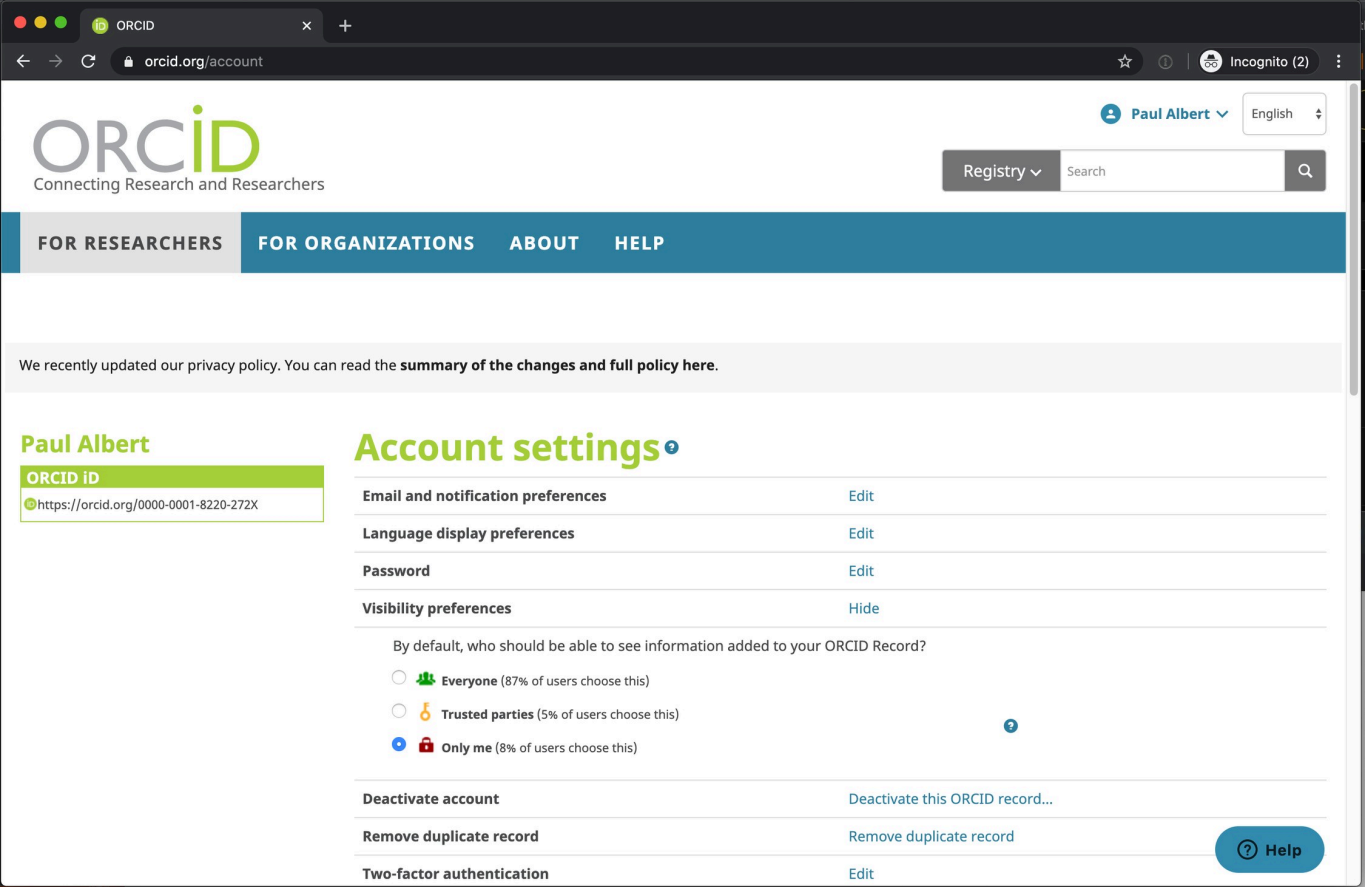
Account settings in ORCID. ORCID users may opt to hide the content of their profiles to ORCID site visitors and data consumers. According to the ORCID website, 13% choose this option.

To address this need, institutions may pay vendors, or rely on librarians or administrators to curate these lists. Most recently, the ORCID author identifier has been widely touted as an effective solution to the problem of mapping papers to people [12], however, this approach has several problems. For one, ORCID identifiers are inconsistently input by scholars at the time of publication: fewer than 6% of Weill Cornell Medicine (WCM) papers in PubMed have an ORCID asserted. See **Fig 1**. Of these papers published in 2019, only 22% or 1 in 5 authors has an ORCID represented. In addition, many scholars have duplicate ORCID identifiers. Also, scholars can log into ORCID and opt to set all their publications to private, which effectively renders their profile a “stub record” or record with limited information or utility. See **Fig 2**.

Profiles RNS [8] is one of the few publicly available authorship prediction algorithms that uses at least some institutionally-maintained identity data while also having the virtue of being used by several institutions in a production environment. This service accepts as input and uses some identity data: name aliases, email addresses, PubMed identifiers (PMIDs) to add and exclude, and affiliation keywords. With these data, Profiles RNS returns as XML a list of PMIDs that have a confidence score exceeding a certain value.

Reviewing the literature, we failed to locate publicly available data sets including among those described by Müller *et al*. [9] that make scholar identities available at anywhere near the richness or potential of those maintained by academic institutions.

## 2. Methods

### 2.1. Identity-driven authorship prediction

It is surprising that mature software is not more widely available or used to solve institutions’ need to maintain publication lists at scale. A given institution may be responsible for tracking thousands of publications by thousands of scholars. In addition, scholars frequently adhere to semi-predictable patterns in their record of scholarship, with some co-authoring a hundred or more papers with a certain colleague and others publishing almost exclusively in a certain niche journal or discipline, or on a particular topic.

Any software that predicts article authorships would do well to emulate certain cognitive subroutines of a savvy librarian who is charged with identifying all of that scholar’s publications and has access to significant portions of a *curriculum vitae* to do so. Librarians generally conclude that a person who received a Bachelor’s degree in 2010 is unlikely to have authored a scholarly paper in 2008 and even far less likely to have authored one in 1998. They know that social workers tend not to contribute to bioinformatics journals. They realize that an article written by an author with a first name of “Terry” is unlikely to be written by a scholar named “Terrie” despite there being a Levenshtein distance of only two between those names. Our shrewd librarian also realizes that students are unlikely to have authored a corpus of several hundred publications while still in medical school, and that the more common a scholar’s name is, the less likely that person is to have authored any one candidate paper. Further, they know that when even one of a group of closely related articles was (or was not) written by a scholar of interest, we can more accurately predict whether the other articles were.

**Fig 3:**
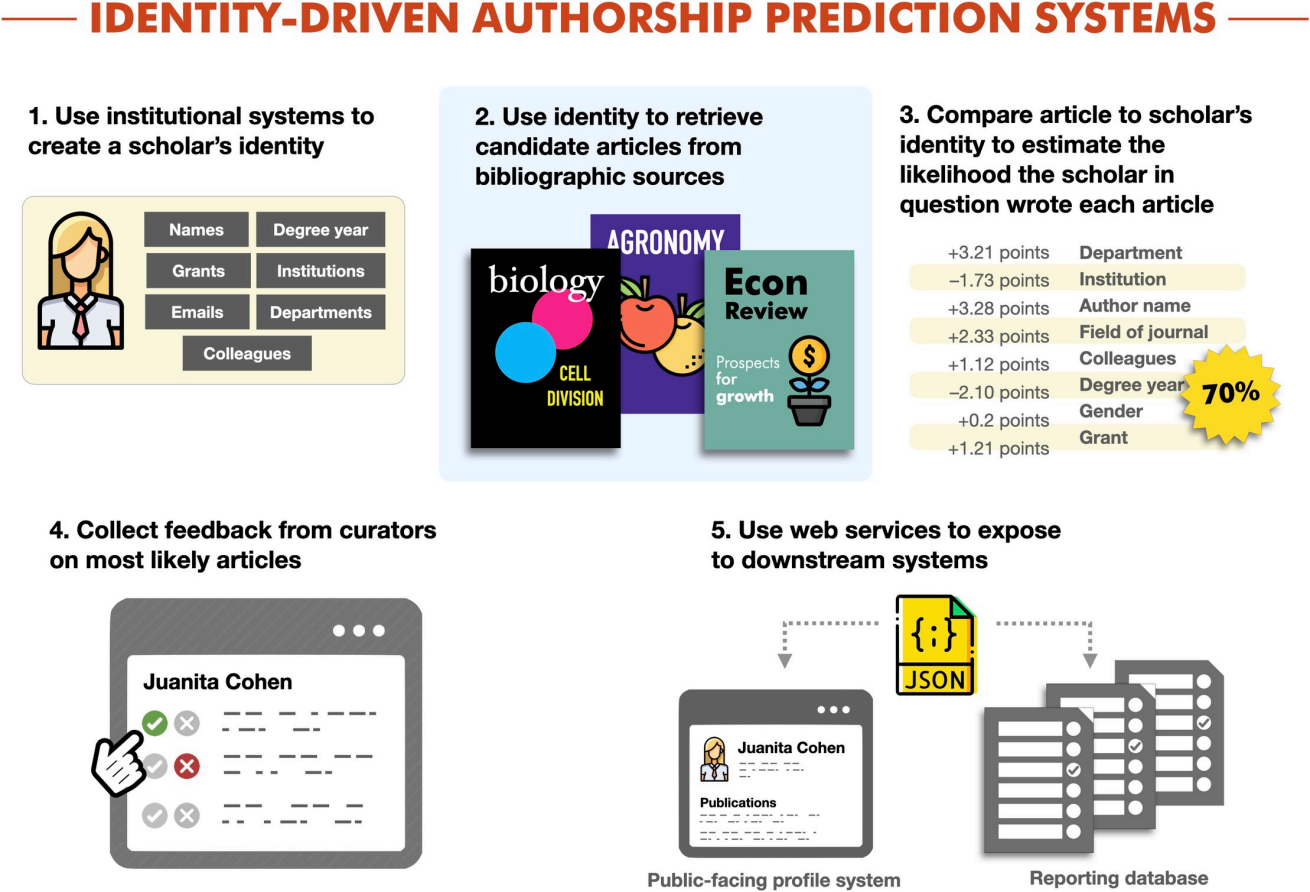
Identity-driven authorship prediction systems. Systems that leverage institutionally available information about a scholar to retrieve candidate articles and predict which of those articles a scholars has written. Curators provide feedback on those predictions, and data is shared with downstream systems.

The process of using software to predict publication lists for scholars necessarily starts with a critical mass of information about the scholars themselves. These person metadata include name aliases, email addresses, names of grants, departmental affiliations, institutional affiliations, and names of colleagues who are potential co-authors. While these data are available in a *curriculum vitae*, they are also stored and regularly updated in many academic institutions’ information systems. We define identity-driven authorship prediction as the process of using information about a scholar, as recorded outside of a bibliographic system, to quantify the likelihood that person wrote a set of articles. See **Fig 3**.

Many human-facing algorithms are opaque, failing to explain, for example, why a particular book, movie, or restaurant was recommended.

Qian *et al*. write [10] that human judgment purely on the basis of algorithms is not suitable because the automatically produced results are not understandable for humans. This is problematic in the case of authorship prediction systems. Even the most advanced algorithm is not 100% accurate, and, at many institutions, the process of maintaining publication lists is the responsibility of departments, librarians, and administrative staff. Absent a clear indication such as a matching email address or ORCID identifier, both of which are infrequent, third parties may not easily recognize an article as being authored by a given scholar. Therefore, it is important that an authorship prediction system plainly explain why a candidate article has been scored as it has as well as provide a notion of relative confidence.

It is likewise practical to track which publications a scholar has not authored both to improve accuracy and to avoid the inevitable questions as to why a particular candidate article is not among the accepted articles.

### 2.2. Approach used by ReCiter

In this article, we describe ReCiter [11], an open source, identity-driven authorship prediction algorithm. ReCiter retrieves a set of candidate articles from PubMed and, optionally, Scopus (standard institutional license required); clusters those articles; and uses the wealth of institutionally-maintained identity data available to academic institutions to score candidate articles. Candidate articles are output with key contextual information, including evidence, which speak to the probability a given scholar wrote a particular paper and allow a third party like a librarian to quickly decide if an article was authored by a scholar of interest.

ReCiter’s approach is distinct from those pursued by many disambiguation algorithms (e.g., Louppe *et al*. [12]) in that these applications are largely geared towards creating clusters of all co-authors. If a scholar has potentially written two papers, and both have five authors each, these systems will attempt to create groups out of the ten authors. ReCiter does perform clustering, but that is a relativvely minor subprocess. Also, with ReCiter, the number of potential matches is the number of articles and no more. This design decision is guided by the needs of consumers of our data who are ambivalent as to whether those ten co-authors ultimately represent ten people or five; they only want to know if the scholar in question wrote neither, one, or both of those articles.

**Fig 4:**
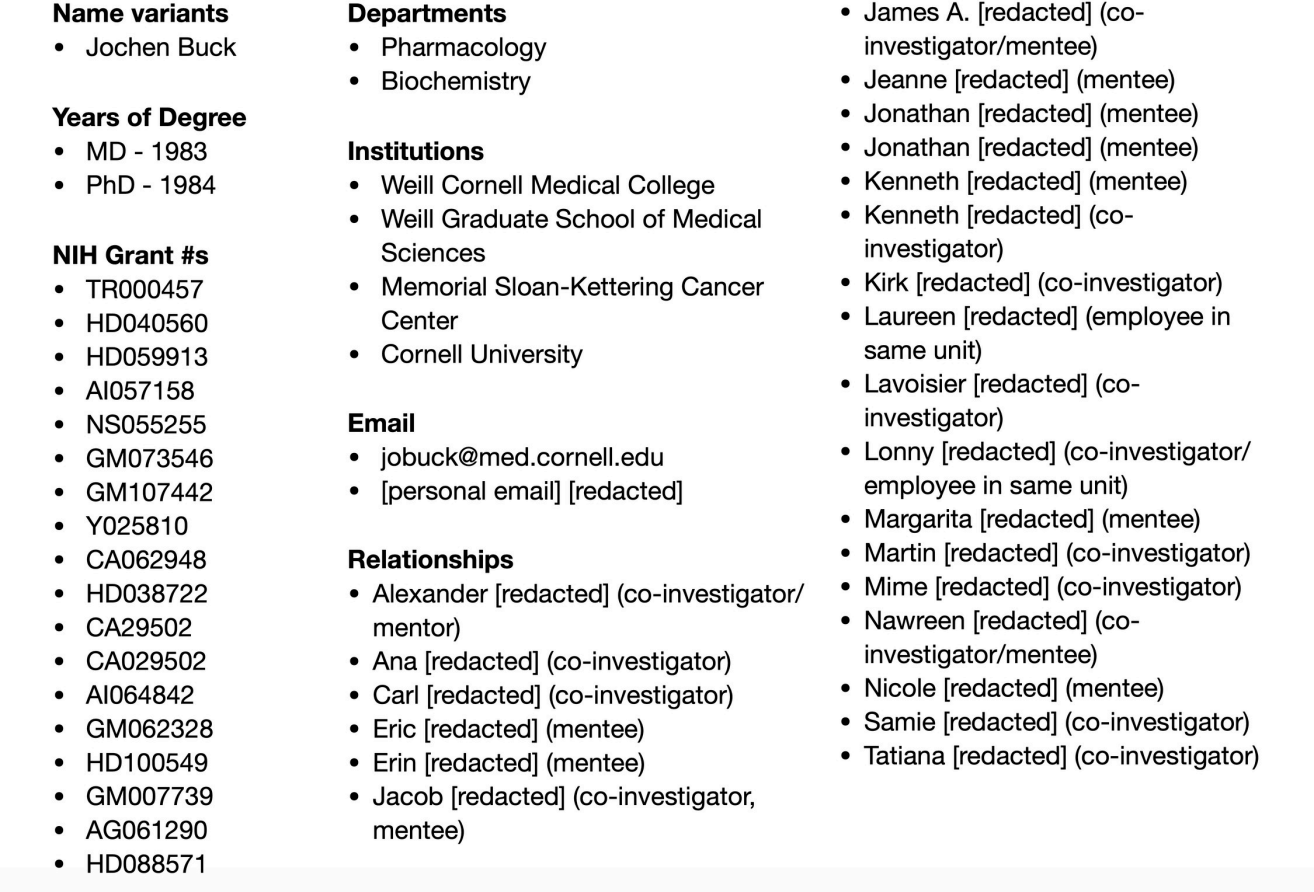
Institutionally maintained metadata about a sample WCM scholar that can be used for programmatic authorship prediction. Identity sources at academic institutions like Weill Cornell Medicine can provide a range of data. These data can be used to programmatically compute the likelihood a given article was written by a given scholar. For example, any known “relationships” are often the names of potential co-authors.

The goal for ReCiter is to fully leverage feedback for whether a scholar wrote an article as well as the abundance of identity information institutions commonly maintain in various systems of record. Useful data includes (see **Fig 4**) a person’s past and present departmental, institutional, and program affiliations; email addresses; years of bachelor and doctoral degrees; inferred or preferred gender; grant identifiers; and the names of known relationships such as mentors, managers, co-investigators listed on grants, mentees, and individuals who share the same Human Resources-maintained organizational unit. Because this is an identity-driven approach, all such information can be employed to predict publications before the system has any confirmation that a scholar of interest has authored a paper. ReCiter outputs: the full citation of a candidate, or potential, article; up to 13 scores, only one of which is the average score of articles in its cluster; and the corresponding evidence for why an article was suggested. ReCiter’s conclusions are not derivative of any other service including ORCID ID, Scopus Author Identifier, etc.

### 2.3. Algorithm

**Fig 5:**
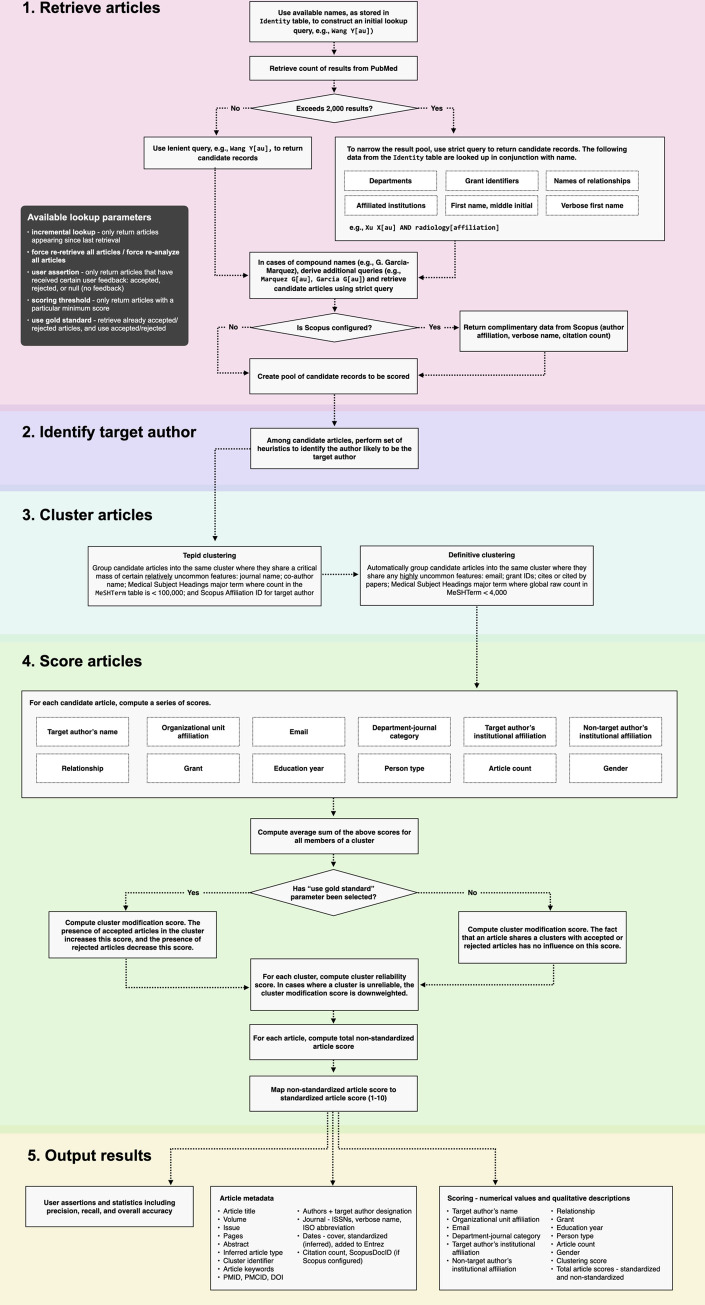
Overview of the ReCiter algorithm. The Feature Generator API coordinates the following key steps in the ReCiter workflow: retrieve candidate records, identify target author, cluster articles, score articles, and output results.

ReCiter identifies, retrieves, and scores candidate records in PubMed for an individual scholar. This entire process is coordinated by ReCiter’s Feature Generator API. The Feature Generator API negotiates with other ReCiter APIs including the ones for identity retrieval, PubMed retrieval, Scopus retrieval (if one so designates), and Gold Standard retrieval. With Gold Standard API, one can download a list of identifiers of articles that have been accepted and rejected by a user. While one can interact with these APIs individually, it is a best practice, at least in a production environment, to allow the Feature Generator API to do this. Feature Generator API can output article metadata, scoring, and qualitative descriptions providing a rationale for scoring (e.g., a candidate record is indexed with a grant matching one in the Identity table). See **Fig 5** for an overview of the algorithm’s workflow and **S1 Appendix** for a sample output of this API.

Search queries are constructed by using the name(s) recorded in the Identity table in DynamoDB. The standard syntax for a name search consists of a verbose last name and first initial, e.g., Albert P[au]. ReCiter will also search for additional candidate records in cases where a scholar has multiple name aliases or a compound surname. For example, the scholar, Gabriel Garcia Marquez, would be searched as Marquez G[au] OR Garcia G[au]. This approach identifies potential articles where scholars may have a maiden name or their middle name is accidentally comingled with their surname, which sometimes occurs in the case of Arab and Latin names. If a user has different names in identity source systems (e.g., one captures a maiden name, another does not), ReCiter will look up each of those names independently.

If the count of candidate records exceeds a set amount–the current default is set to 2,000 –ReCiter does not retrieve any records and instead goes into its “strict” mode to narrow down the result pool. In strict mode, the standard last name, first initial search is made in conjunction with each of six additional search strings. These search strategies are: searching by verbose first name, first and middle initial, names of relationships, grant identifiers, departments, affiliated departments, and affiliated institutions. For example, one of the six strict searches for a scholar named “Xu Xu”, who is in the department of Radiology would be: Xu X[au] AND Radiology[affiliation]. ReCiter also uses strict mode against inferred last names. If a scholar’s last name has a space or dash (e.g., Garcia-Marquez, Garcia Marquez), the system will look up "Garcia" and "Marquez" independently but paired with the additional search strings. This mode is known as STRICT_COMPOUND_NAME_LOOKUP.

By default, all candidate articles have a user assertion of null, which reflects the fact that no human curator has asserted whether the article should in fact be assigned to the scholar of interest. A user can provide feedback to accept or reject an article suggestion. They can also revert previously accepted or rejected articles to null. ReCiter records user feedback including accepted and rejected articles in its GoldStandard table. If the user or agent requesting the lookup for a scholar selects AS_EVIDENCE for the use-gold-standard parameter, records from the GoldStandard table are also retrieved. If FOR_TESTING_ONLY is selected, ReCiter will only retrieve and score articles retrieved by the name lookup.

Once a pool of candidate articles is retrieved, additional data about a given article are optionally retrieved from Scopus by doing a DOI search against its API. These data include Scopus institutional identifiers, verbose author name, and citation count.

ReCiter uses metadata from PubMed and Scopus to output a single canonical publication type for each article, and also outputs a standardized/sortable date of publication and a display date. For the purposes of clustering and scoring, ReCiter will standardize all names, removing suffixes, dashes, and diacritical marks such as accents.

For every retrieved candidate article, ReCiter uses a set of heuristics to identify the author who is most likely to be the target author. Our approach has some overlap with the heuristic proposed by Torvik and Smalheiser [13]. According to our analysis, our system is able to identify the correct target author more than 95% of the time. The correct assignment of target author, even in cases of a true negative, can be quite helpful for accurately scoring the candidate articles. Clustering uses target author assignment as do six of the scoring strategies. It is also useful for business reporting. The Office of Research asks these authors to see all the papers in the prior month appearing in “top” journals where a principal investigator was senior author.

In the clustering stage, ReCiter uses an unsupervised rule-based agglomerative algorithm to create groups of articles written by the same person. There are two steps to clustering: “tepid clustering” and “definitive clustering.”

With tepid clustering, we identify features, which are somewhat uncommon (frequency of around 100,000 records or fewer out of a corpus of 40+ million). The features used in tepid clustering are: journal name; co-author name excluding cases where an author is identified as a target author and extremely common co-author names [13, 14]; Medical Subject Headings (MeSH) major term where count in the MeSHTerm table is < 100,000; and Scopus Affiliation ID for target author. By themselves, these features are not positively conclusive. But, if a certain proportion of features between any two articles share these features, we can conclude they were written by the same person, and they are clustered.

Clusters are merged if the cluster-cluster-similarity-score-threshold, as stored in application.properties, is less than the computed value based on raw counts and overlap from those clusters:

cluster-cluster-similarity-score < (cluster1 ⋂ cluster2)^2^ / (count-of-items-in-cluster1 * count-of-items-in-cluster2)

For example, suppose there are two clusters:

{

          Cluster Id: 1

          Journals: Cell;

          MeSH major: Thalassemia, Sunlight, Tamoxifen, Tryptophan, Brain;

          Coauthors: Marshall T, Michaels A;

          Scopus institutional affiliation identifiers for target author: 6007997;

}

{

          Cluster Id: 2;

          Journals: Cell;

          MeSH major: Thalassemia, Tamoxifen;

          Coauthors: Marshall T, Michaels A, Johnson Q;

          Scopus institutional affiliation identifiers for target author: 6007997, 342823053;

}

The system would conclude these clusters should be merged as 0.2 < (6^2) / (9 * 8). The clustering is designed to work even if Scopus affiliation identifiers are not available.

With definitive clustering, we look to combine clusters where features generally occur thousands or fewer times in a corpus of 40 million records. Because they occur so infrequently, we will merge clusters whenever there is a single such piece of evidence. Any article that shares any of these features with another article should be in the same cluster as that other article: email; grant identifiers excluding papers that mention more grants than listed in a threshold in application.properties; cites or cited by papers; MeSH major where global raw count in MeSHTerm < 4,000.

Then, the candidate articles are individually scored. ReCiter is object-oriented and uses a strategy design pattern [15] in which each evidence type is modeled as a different strategy and appears in a separate module within ReCiter. In the first round of scoring, a given candidate article is scored using 12 criteria, which we hypothesized are correlated with whether an article would be accepted or rejected.

- **target author’s name**—similarity of name recorded in the Identity table compared to target author’s name
- **organizational unit**—whether any departments, divisions, etc. in Identity appear in the affiliation statement of the target author
- **email**—if the email in Identity appears in a target author’s affiliation statement
- **department-journal category—**a score that reflects the extent to which a scholar’s organizational unit (e.g., Orthopedic Surgery) is highly associated with the ScienceMetrix category [16] (e.g., Orthopedics) of the journal
- **target author’s institutional affiliation**—if affiliation recorded in Identity is consistent with affiliation listed in the author affiliation statement for the target author
- **non-target author’s institutional affiliation**—if affiliation recorded in Identity is consistent with affiliation listed in the author affiliation statement for any author *except* the target author
- **relationship**—if individuals known to have worked with a scholar, including those who are listed on a shared grant or have served as a mentor, match any co-author names
- **grant**—if a candidate article is indexed with an NIH grant identifier recorded in the scholar’s Identity profile
- **education year**—effect scholarly age has on the likelihood an article in a given year was written by a scholar; especially penalizes candidate articles that are published far earlier than expected; works on a sliding scale
- **person type**—factors into account how often different types of scholars (e.g., faculty, postdoc, medical student, etc.) are likely to author an article
- **article count**—a score for how many candidate articles were identified for a given author; score increases when there are fewer candidate articles and decreases when there are more candidate articles
- **gender—**a score, which uses Social Security Administration data [17], to infer the likelihood a name in the Identity table shares the gender with that of the name of the target author

In some cases such as email-match-score, the score is a constant stored in ReCiter’s configuration file, application.properties. With the "article count" evidence, consistent with Bayesian insights about probability, we reward individual candidate articles, in which there are few articles and penalize cases in which there are a lot. We do this for each article using three values:

- count-articles-retrieved–count of the articles actually retrieved
- article-count-threshold-score–as stored in application.properties, e.g., 800
- article-count-weight, as stored in application.properties, e.g., 200

The value for article-count-score for each article is equal to:

(article-count-threshold-score–count-articles-retrieved) / article-count-weight

Here is sample output for a scholar’s ("C. Cole"), article count score, where the scholar has 1,151 candidate articles:

"articleCountEvidence": {

    "countArticlesRetrieved": 1151,

    "articleCountScore": -1.755

},

In other scores, the scoring logic is more complicated. The score for a target author’s name is the sum of individual subscores for first name, middle name, and last name. In scoring first name, the algorithm attempts to identify which of the 10 types of match between name metadata in the Identity table and the given name of the target author is highest. Matches that are more tenuous have lower scores. For example, the strongest match would be full verbose name (“Terrie” vs. “Terrie”). Less strong matches include those based on inferred initials (“Paul J.” vs. “PJ”), a fuzzy match (“Michael” vs. “Michel”), and a case where initials are present but in an unexpected order (“JD” vs. “DJ”). The weakest scores correspond to cases where names conflict partially (“Ximeng” vs. “Xu”) or entirely (“Curtis” vs. “Ronald”). The algorithm also looks for various other cases where first and middle name may be inverted in the article metadata or middle name may be combined with last name. **S2 Appendix** describes this logic in greater detail.

We then compute cluster-score-average, the average score of all articles in a cluster. If the user requesting the lookup for a scholar selects AS_EVIDENCE for the use-gold-standard parameter, the presence of accepted articles in a cluster increases the cluster-score-average by a value set in application.properties while the presence of rejected articles decreases this value. Conversely, when the FOR_TESTING_ONLY parameter is selected for use-gold-standard, there is no such effect.

If the cluster-score-average is higher than that of the target article (total-article-score-without-clustering), the target article’s total score increases. If it is lower, it decreases. We also compute a cluster-reliability-score, in which ReCiter gauges the extent to which a target author’s verbose first name, where it exists, is consistent within a given cluster. If it is inconsistent (e.g., a given cluster contains articles with target authors that have the first name of RockBum, RaeKwon, RulBin, and RyoonHo), we downweight this effect. The sum of all the different evidence subscores is total-article-score-nonstandardized, a value which is mapped to a standardized score (total-article-score-standardized) between 1 and 10.

The Feature Generator API allows users to choose whether to restrict article retrieval to those added since the last time ReCiter retrieved articles for a scholar. Also, administrators can elect whether to return articles of a particular status (e.g., “accepted”, “rejected”, “null” in which any feedback has yet to be provided, or some combination thereof) and which have a minimum score.

For each article, ReCiter outputs one of four designations as seen in **Table 1**.

**Table 1:** Significance of accuracy designations in Feature Generator API output.

	Computed total standardized article score (1–10) is greater than or equal to threshold (1–10) supplied by user	Computed total standardized article score (1–10) is less than threshold (1–10) supplied by user
User feedback as recorded in GoldStandard table is ACCEPTED	True Positive (TP)	False Negative (FN)
User feedback as recorded in GoldStandard table is REJECTED or NULL	False Positive (FP)	True Negative (TN)

The following scholar-level measurements of accuracy are computed and output:

precision = TP / (TP + FP)

recall = TP / (TP + FN)

accuracy = (TP + TN) / (TP + TN + FP + FN)

Measurements of accuracy can depend on several factors. Name origin is widely discussed in the literature, but academic age is especially important for algorithms that perform authorship prediction. We could test ReCiter’s accuracy only by using longstanding faculty at Weill Cornell in cases where we have a rich set of identity data and a lot of publications to show relationships. In such cases, ReCiter may perform relatively well. For those scholars early in their careers, the algorithm is generally less forgiving.

### 2.4. Evaluation

#### 2.4.1 Creating and using a support vector machine-based training model

The original set of weights used to compute the 13 scores for any given candidate article were no more than the product of developer intuition, and trial and error. To improve the accuracy and overall performance of ReCiter we sought to optimize these constants. To do so, we randomly selected a group of 500 Weill Cornell-affiliated scholars, which consisted of full-time WCM-employed faculty (n = 310), affiliated faculty (n = 82), postdocs (n = 68), and third- or fourth-year M.D. students (n = 40). We purposefully targeted a heterogeneous group including types of scholars such as M.D. students who are unlikely to have authored any more than several publications. If our goal was to maximize accuracy for this analysis, we could have limited ourselves to only principal investigators and other well-established senior scholars. Our approach at least partially guards against the possibility that ReCiter could be overly aggressive in suggesting publications. It also tests the accuracy of an authorship prediction system in cases when there is sparse identity data for a scholar, and there are fewer signals to leverage. We subdivided candidate articles for our 500 individuals of interest into a training group and a test group. The training group represented 80% of all candidate articles, and test group represented 20% of all candidate articles. For privacy concerns, we are not able to share the identifying details of the 500 selected scholars without securing permission from each of them. However, we do want to inspire some confidence that these names have not been selected in a self-serving way. **S3 Appendix** includes the surnames and first initials of the 86 scholars who have the most common names as defined by anyone who had 2,000 or more candidate articles.

ReCiter estimates the likelihood a given scholar authored a given article by using personally identifiable information including personal email, names of mentees, etc. As such, it is not possible for us to share the raw data.

To create our gold standard of known publications, we started with any assertions about author identity for our 500 individuals available in our legacy system. Starting in 2014 to the present, the publications authored by full-time WCM-employed faculty, affiliated faculty, and postdocs, have been recorded and updated in an institutionally maintained MySQL database containing approximately 185,000 publications. Data from this system is repurposed in a variety of forums and has received feedback through a number of touchpoints. Publication data are published on users’ public-facing VIVO scholarly profiles. They appear in T32 training grants applications [18] and in reports for the Dean, Office of Research, department chairs, Office of External Affairs, Institutional Reporting, and various educational program offices. When full-time faculty start at Weill Cornell Medicine, librarians manually curate lists of all these individuals’ publications. Missing or incorrectly assigned articles frequently elicit feedback sent to the Library and are manually corrected in that database. All full-time WCM-employed faculty are required to review their publications on an annual basis. This feedback may include group authorships (e.g., “HI-TECH Investigators”) in which there is no indication other than an individual scholar’s assertion that a given scholar contributed to a paper. Publications authored by MD students were reviewed by the Office of Medical Education.

All assertions were manually reviewed by two librarians who updated publications lists for all individuals between October 28 and November 21, 2019. A second librarian identified 45 articles that were incorrect. Of these, four were reverted. Any candidate articles that were not accepted were implicitly asserted to be rejected.

Of the 16,330 publications ultimately judged to be authored by our 500 scholars of interest, 79.8% were originally recorded as such in the legacy reporting database. This figure increases to 88.1% when only considering the 310 in scope full-time faculty. We attribute this higher figure due to the greater level of scrutiny in maintaining publication lists for full-time faculty. By comparison, publications authored by affiliated faculty are typically added only by explicit request. While some of the discrepancies between the two systems are the result of curation errors, especially errors of omission, a non-trivial portion of the differences are the result of Scopus incorrectly crosswalking records from PubMed to Scopus. This is particularly common in the case of comments and other types of publications that refer to another publication.

For all in-scope individuals, we used a Bash script (see **S4 Appendix**) to query ReCiter’s Feature Generator API and output the result including article metadata, evidence, and scoring to a series of JSON files. For the use-gold-standard option, we selected the FOR_TESTING_ONLY parameter. Typically, the Feature Generator API is set to only store articles of a minimum score (this cuts down on storage costs by approximately 85%), so we could not export data directly from our Analysis table. We then used the ML_Model_Test_upload.py (see **S5 Appendix**) Python script to transform the JSON files into individual flat comma-separated values (CSV) files, which can be loaded into a database (see **S6 Appendix**).

We considered and experimented with several possible machine learning approaches to optimize the weights of the constants in the application.properties configuration file: random forest, linear regression, and support vector machine. Because our data set has far more rejected articles than accepted articles, we opted to use a support vector machine (SVM) analysis [19], which has a reputation for being more robust in the presence of unbalanced data. SVM can produce results that are explained when using a linear kernel, and we can incorporate the model results into the original linear regression system. Also, it is reputed to be a strong choice for performing binary classification [20]. All the following operations are included in a Jupyter notebook, available in **S7 Appendix**.

In order to use weights calculated by the model, we used Linear SVM [21]. This library fits the dataset and generates a hyperplane for dividing the data into two groups: those articles the algorithm concludes were written by the scholar of interest and those the system concludes were not. Using Linear SVM, we can also identify the weight and intercept of this hyperplane in the model output, and infer the importance of each feature. By using a decision function, which is calculated based on weight and intercept, we are able to predict confidence scores for samples. The confidence score for a sample is the signed distance of that sample to the hyperplane. With a higher absolute value of confidence score, we are more certain with the prediction of the sample.

Confidence score was computed using the below equation, in which X_test1[i] is candidate articles’ feature score, clf.coef_[0] are the weights generated by the model, and clf.intercept_[0] is the intercept generated by the model. The np.matmul function is the sum of the features multiplied by their respective weights.

confidenceScore=np.matmul(X_test1[i],clf.coef_[0])+clf.intercept_[0]

It is generally preferable to first normalize data and then feed it into an SVM model. But in our case, we did have an *a priori* assumption of the importance of features, so we concluded that it would be acceptable to preserve the original scale. Also, we experimented with tuning the regularization parameter of the SVM model, with values from 0.0001 to 100, increasing by a multiple of 10. We determined that the regularization parameter of 1 provides the optimal value.

In order to test the robustness of the parameter weights output by SVM, we used cross-validation. Additionally, we tried the Pearson Correlation and Random Forest feature selection methods. With Pearson Correlation, we computed and output the correlation between each feature to determine if any features were so highly correlated we could conclude they may be redundant. With Recursive Feature Elimination, we used SVM as a model. Finally, we experimented using Random Forest and looked at the embedded feature importance. We tried to adjust the weight generated from SVM manually by incorporating the feature ranking from all of these three models, but these changes did not improve the system performance, so we only used the weight generated by Linear SVM.

We updated the weights in the application.properties file (see **S8 Appendix**), multiplying the existing values by the values output in this exercise. We then re-ran ReCiter for all candidate articles in our test set. We then sampled the test set and compared the new system’s performance with the old one.

We produced two different approaches to evaluate the system’s ability to predict scholars’ publications. The first was to calculate performance of all candidate articles for all 500 scholars. In this method, articles are pooled prior to analysis. With the second approach, performance is first computed by individual person and results are then averaged. In both cases, we choose a threshold value for total-article-score- nonstandardized that would yield the highest overall accuracy. Going forward, except where otherwise indicated, we will use the term, "article score" synonymously with total-article-score-nonstandardized.

#### 2.4.2. Optimization of standardized score intervals

ReCiter outputs two overall scores for a given candidate article: a raw score and a standardized score. The standardized score is the result of mapping the raw score to a set of ten intervals on a scale of 1–10. So long as these intervals are updated appropriately, we are free to update the weights that comprise a given article’s raw score while still maintaining a relatively consistent and expected distribution of scores. Our goal is that an end user would be able to develop over time a reliable instinct for what it means for an article to have a standardized score of, say, 8.

After completing the SVM-based optimization of evidence weights, we took the raw scores for all candidate articles for all our in-scope scholars and computed the percentile for each article. For each percentile, we computed the percentage of articles that have been accepted. Over this graph, we computed a trendline, which was a simple moving average. This allowed us to graph the relationship between article score and likelihood that a given article has been accepted.

## 3. Materials

The first version of ReCiter was developed by two of us (S.J. and M.B.) in 2010 in object-oriented Perl and output results in a text file [22]. Also, it focused on author name disambiguation, as we rigorously define it above, as opposed to authorship prediction. For that prototype version, we evaluated ReCiter and found its disambiguation accuracy to be around 89%, which was comparable at the time to the accuracy in Scopus. Since then, the original code has been completely re-written in Java, migrated to Amazon Web Services, and reoriented towards the authorship prediction problem. Through a number of changes to the code and its scope, ReCiter has been modified to facilitate adoption by academic institutions.

**Fig 6:**
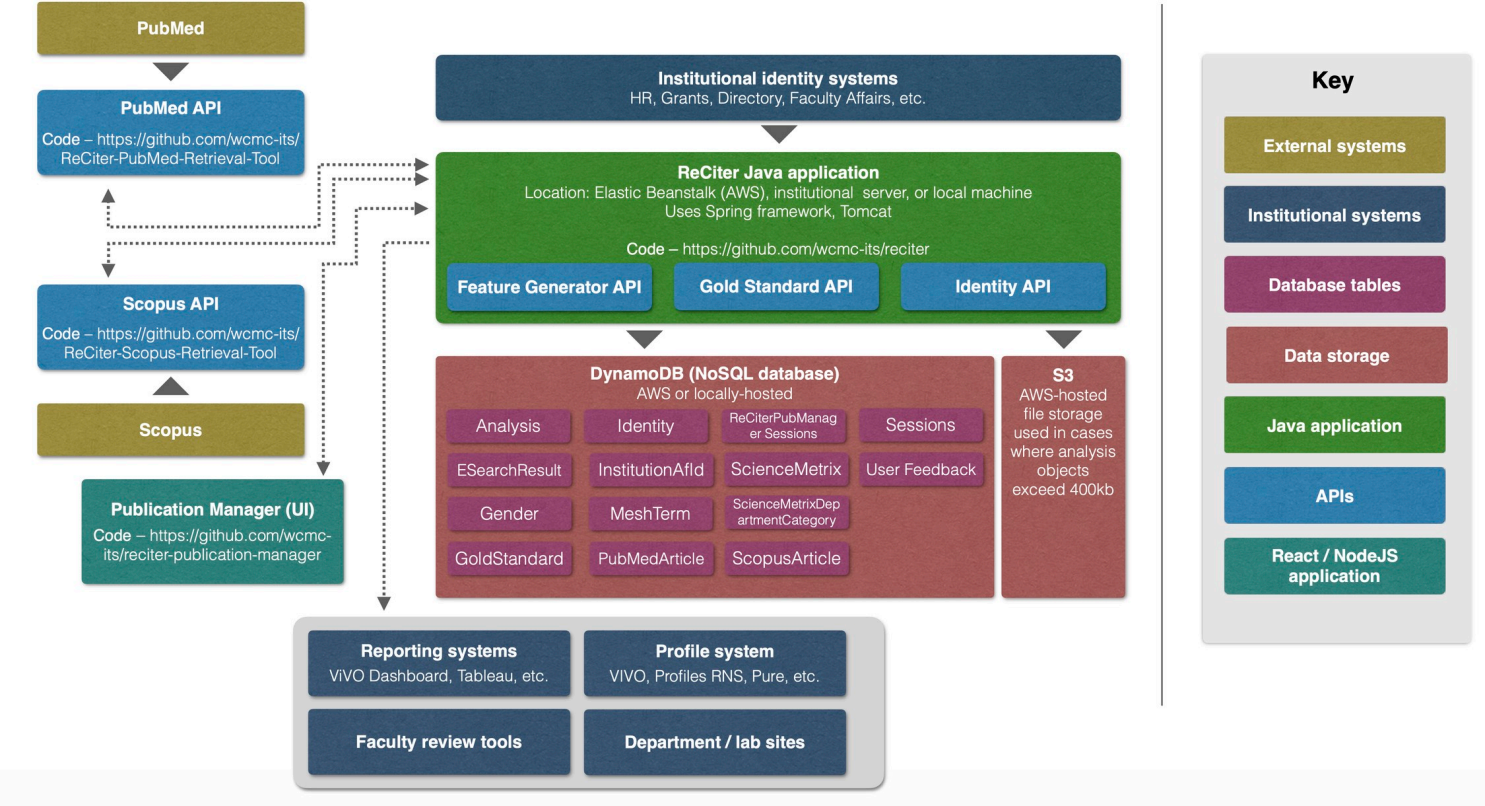
ReCiter architectural diagram. ReCiter contains several components including ReCiter itself, the PubMed Retrieval Tool, the Scopus Retrieval Tool, and ReCiter Publication Manager. Each of these components can be used independently. Identity data is fed from institutional sources. Articles are retrieved from the PubMed API and, optionally, the Scopus API. The ReCiter application computes scores and shares suggestions through a set of web services.

ReCiter is a suite of four applications, each of which can be used as a standalone service: ReCiter, the ReCiter PubMed Retrieval Tool, the ReCiter Scopus Retrieval Tool, and ReCiter Publication Manager. (See **Fig 6**) These applications communicate with each other using a growing list of 23 application programming interfaces (APIs) with the data format in JavaScript Object Notation (JSON). These systems also use Swagger [23] as a toolset that provides a self-documenting user interface, offering helpful cues for how to interact with a given API.

The main ReCiter application, including its computation logic, is written in Java and employs Spring, a framework which manages web services and service requests. ReCiter stores all data about researchers and publications in DynamoDB, a NoSQL database. In cases where data objects exceed 400 KB, data are stored in the S3 file storage system. Identity data is loaded at application start via a JSON file or can be added via one of several APIs for loading identity data for a single user or multiple users. All data objects are described by several separate code repositories [24–28] and conform to models controlled in Maven Central [29]. Most institution-specific configuration occurs within the application.properties file [30]. This file, which contains a great deal of inline documentation, is set as a default to several settings that are specific to Weill Cornell Medicine including synonyms for home institution, names of collaborating institutions, and various other configuration settings.

Both the National Library of Medicine’s eFetch API and Elsevier’s Scopus API return data in XML. The PubMed Retrieval Tool and Scopus Retrieval Tool take these outputs and transform them into the more developer-friendly JSON format. The Scopus Retrieval Tool removes duplicate authors from the Elsevier feed. The PubMed Retrieval Tool uses a multi-threading strategy known as a work stealing pool [31] to allow for up to ten retrieval requests at a time. In cases where the PubMed Retrieval Tool fails to return a record because of downtime or a bug, it will try again up to nine times, with a delay that increases as per a Fibonacci sequence delay. Both PubMed and any matching Scopus article data are stored in separate DynamoDB tables.

ReCiter has been designed to be easily installed on Amazon Web Services (AWS). A freely available CloudFormation template coordinates the installation of all its dependencies and configuration values, allowing a developer to install a fully working version of ReCiter, with all the above systems on AWS along with continuous integration and deployment, in around 20 minutes. Alternatively, ReCiter can also be run on a local machine or independently-controlled server. Of course, institutions must supply identity information about their scholars. This can be done via a file load imported at runtime or using an API.

## 4. Results

**Fig 7:**
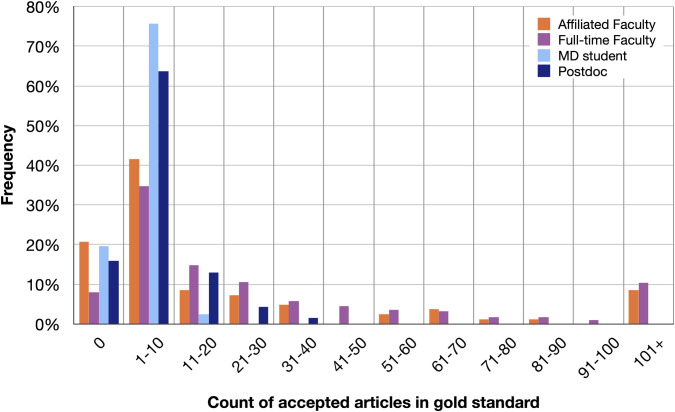
Count of accepted articles by a scholar’s type. Depending on the type of person, between 8% and 21% of individuals in the training set have not authored a single article in PubMed. The presence of such individuals cautions against the practice of being too aggressive in assigning articles to scholars. Only affiliated faculty and full-time faculty have individuals who have authored more than a hundred articles.

In curating the gold standard, we concluded that our randomly selected set of Weill Cornell-based full-time faculty authored a median of 15 articles, affiliated faculty a median of 5 articles, postdocs a median of 5 articles, and MD students a median of 2 articles. The maximum number of articles authored by an individual scholar was 853. The mode for all person types is 0. Some 60 scholars, or 12%, had no articles in the gold standard. This includes six scholars who had no articles in the gold standard and no candidate articles of any kind. For the 60 scholars who had no accepted articles, ReCiter incorrectly suggested a total of 54 articles or 0.9 articles per scholar. See **Fig 7**.

We described above how ReCiter uses “strict mode” to significantly limit the number of candidate records under consideration when a scholar has a common name (e.g., Y. Wang) or a compound name (e.g., G. Garcia-Marquez). For our 500 scholars, ReCiter used “lenient mode” for 397 scholars, a strict compound name lookup for 17 individuals, and strict mode due to exceeding our results threshold on 86 occasions. **S3 Appendix** provides a list of the surnames, first initials, and count of results of the 86 cases where the count of initial search results exceeded the lenient lookup threshold.

The use of strict mode allows the system, for example, to limit the count of candidate records to be scored for a scholar named Yi Wang from 151,000 to 293 and for Ajay Gupta from 12,340 to 1,340. The overall accuracy for all individuals looked up in lenient mode without first aggregating by individual was 98.18% and in strict mode was 98.49%.

0.76% of candidate articles accepted for a scholar were not retrieved. The reasons for why a given article for a given scholar was not retrieved for consideration include: the name of scholar on publication was inconsistent with name recorded in the identity source system (e.g., scholar uses maiden name); scholar was part of a group authorship; or, the search was done in strict mode. For example, the latter can become problematic when the institution has no record of a scholar’s prior affiliations.

**Fig 8:**
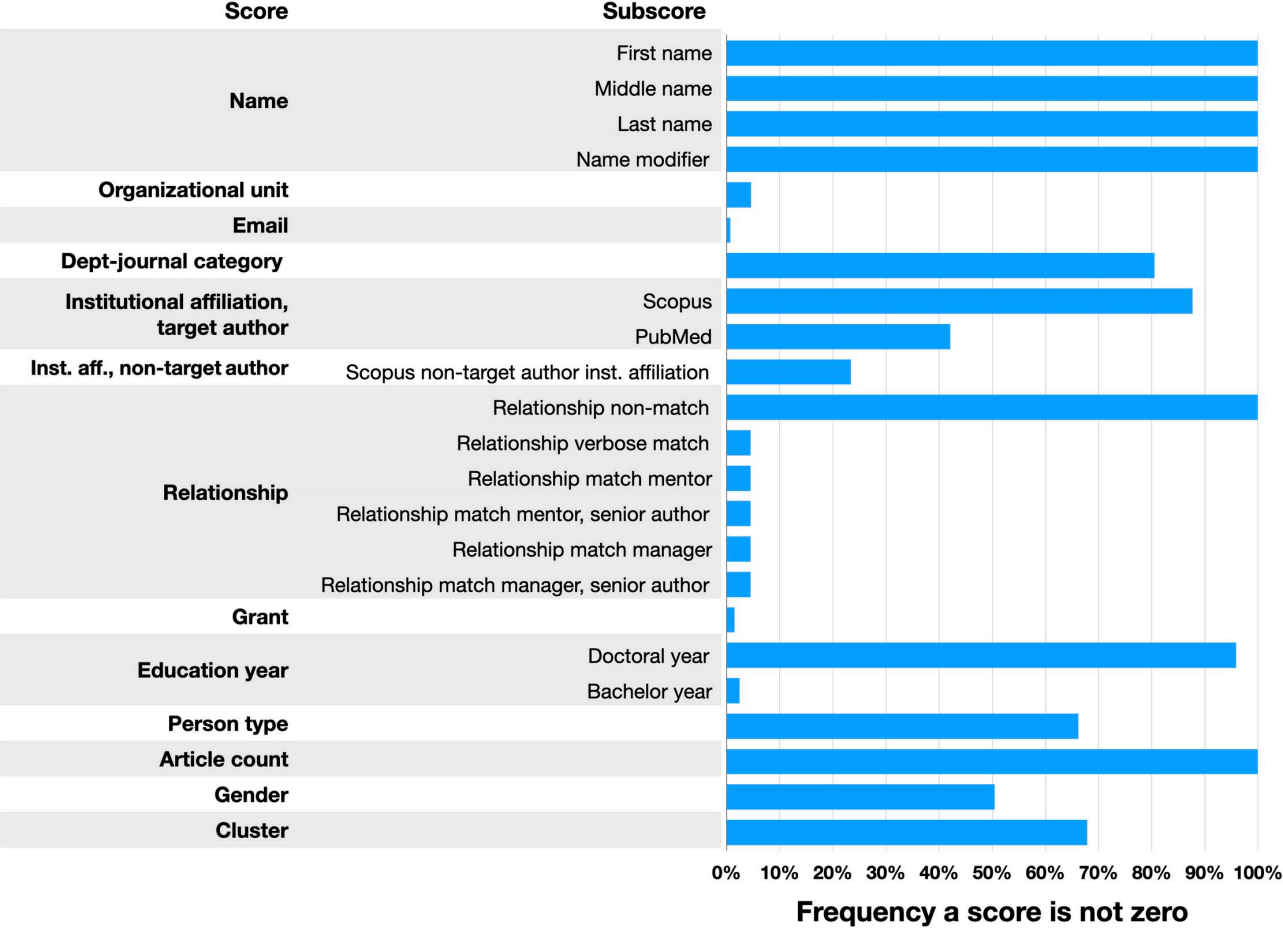
Frequency a score is not zero in the training set. The overall score for a given candidate article is the sum of multiple individual scores (e.g., email score, grant score, etc.). Each of these scores may themselves be the sum of certain subscores, many but not all of which are listed here. For example, to compute name score, we take the name listed in a candidate article and individually evaluate it against any available first, middle, and last name available in the Identity table.

**Fig 8** shows how the different scores are computed and how common certain scores in our training set are not 0. The system’s ability to output a score based on these data for any given article is variable, so it helps to have a rich profile for each scholar. A more detailed explanation of the weights used to create these scores are included in **S2 Appendix**.

We assessed ReCiter’s accuracy according to the equation provided above. We also computed balanced accuracy owing to the fact that this metric is suited for assessing performance of imbalanced datasets [32]. The formula for balanced accuracy is as follows:

balancedaccuracy=(truepositives/(truepositives+falsenegatives)+truenegatives/(truenegatives+falsepositives))/2

We define a true positive or true negative as an article in which the ReCiter algorithm and the judges agreed that the article was, respectively, written or not written, by a scholar of interest. A false positive is an article in which the ReCiter algorithm concluded that the article was written by the scholar of interest, but the judges disagreed. A false negative is an article in which the ReCiter algorithm concluded that the article was not written by the scholar of interest, but the judges disagreed.

There are two approaches for computing the various measures of accuracy. We can either first combine all candidate articles by all scholars into a single pool and then compute accuracy independent of scholar, or compute the accuracy of each individual scholar and then take an average of all the scholars’ scores. Of these two methods for computing accuracy, we prioritize the former as it is a better reflection of the number of times a librarian or administrator will have to provide feedback on erroneous false positives and negatives. In the case of the pooled analysis and where both PubMed and Scopus were used, the accuracy was 98.3%; in the case where accuracy was first computed for individuals, the accuracy was 93.6%. (See **Table 2.)**

**Table 2:** Weights were optimized to maximize accuracy when all articles are first pooled and then accuracy was computed. Accuracy of ReCiter.

	Pool all articles, then compute accuracy	Pool all articles, then compute accuracy	Compute accuracy for individuals, then average	Compute accuracy for individuals, then average
**Data source**	PubMed + Scopus	PubMed only	PubMed + Scopus	PubMed only
Accuracy	0.9826	0.9795	0.9363	0.9231
Balanced accuracy	0.9487	0.9658	0.8505	0.8424
Precision	0.8706	0.8539	0.8299	0.7913
Recall	0.9040	0.9489	0.8824	0.8786

When the only data source used is PubMed, these figures show only a modest drop in accuracy to 98.0% and 92.3%, respectively.

Even so, using Scopus in conjunction with PubMed as opposed to PubMed alone does seem to have a more pronounced effect on the median score of accepted and rejected articles. When using PubMed and Scopus, the median score of accepted articles is 13.91 and rejected articles is -2.23. When using PubMed alone, the median score of accepted articles is 13.01 and rejected articles is -1.87. The gap in scores between accepted and rejected articles is 1.32 greater when using Scopus and PubMed compared to PubMed alone. In some cases, this can be meaningful to the reviewer, changing the standardized score from a 4 out of 10 to a 7. Potentially, a subset of publications would be displayed with Scopus as opposed to without.

**Fig 9:**
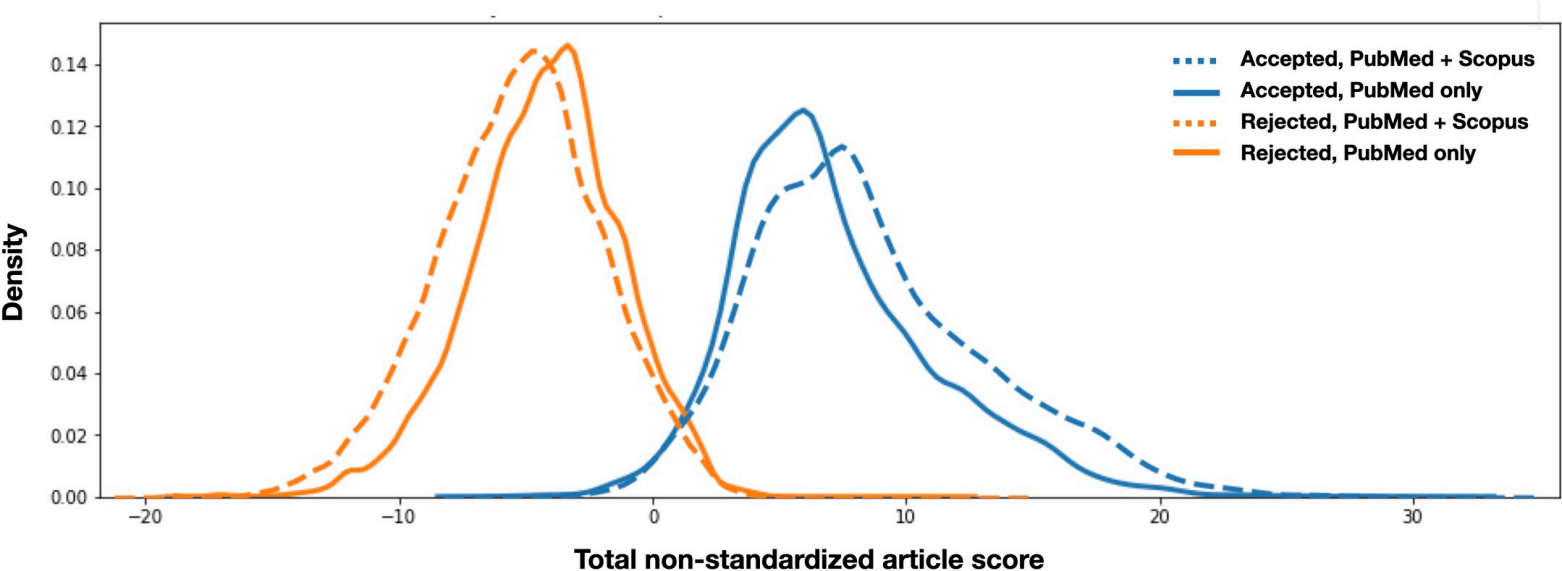
Density graph using PubMed and Scopus versus PubMed alone. Here, the x-axis is the raw score and y-axis is the frequency or density of the total-article-score-nonstandardized. The intersection between the accepted and rejected curves indicates ambiguity. This intersection is largely consistent with or without using complimentary data from Scopus. However, the median difference between accepted and rejected curves is noticeably different in these two scenarios.

**Fig 9** visualizes how common the scores of candidate articles are when all articles are pooled ahead of time using PubMed alone versus PubMed and Scopus combined.

The process by which scores are computed for different types of evidence does not lend itself perfectly to SVM analysis. For example, name score is a subtotal of first-name-score, middle-name-score, last-name-score, as well as odd cases (e.g., middle name is conflated with last name). Nonetheless, the SVM analysis did show us the extent to which certain scores are underweighted or overweighted. One useful insight that emerged from this analysis was how useful it is to match a known relationship to a co-author and to have that co-author match the full verbose first name of that colleague as opposed to just the first initial. Indeed, compared to 3,974 records where such a match occurred in accepted articles, this match was only present in 14 rejected articles.

**Fig 10:**
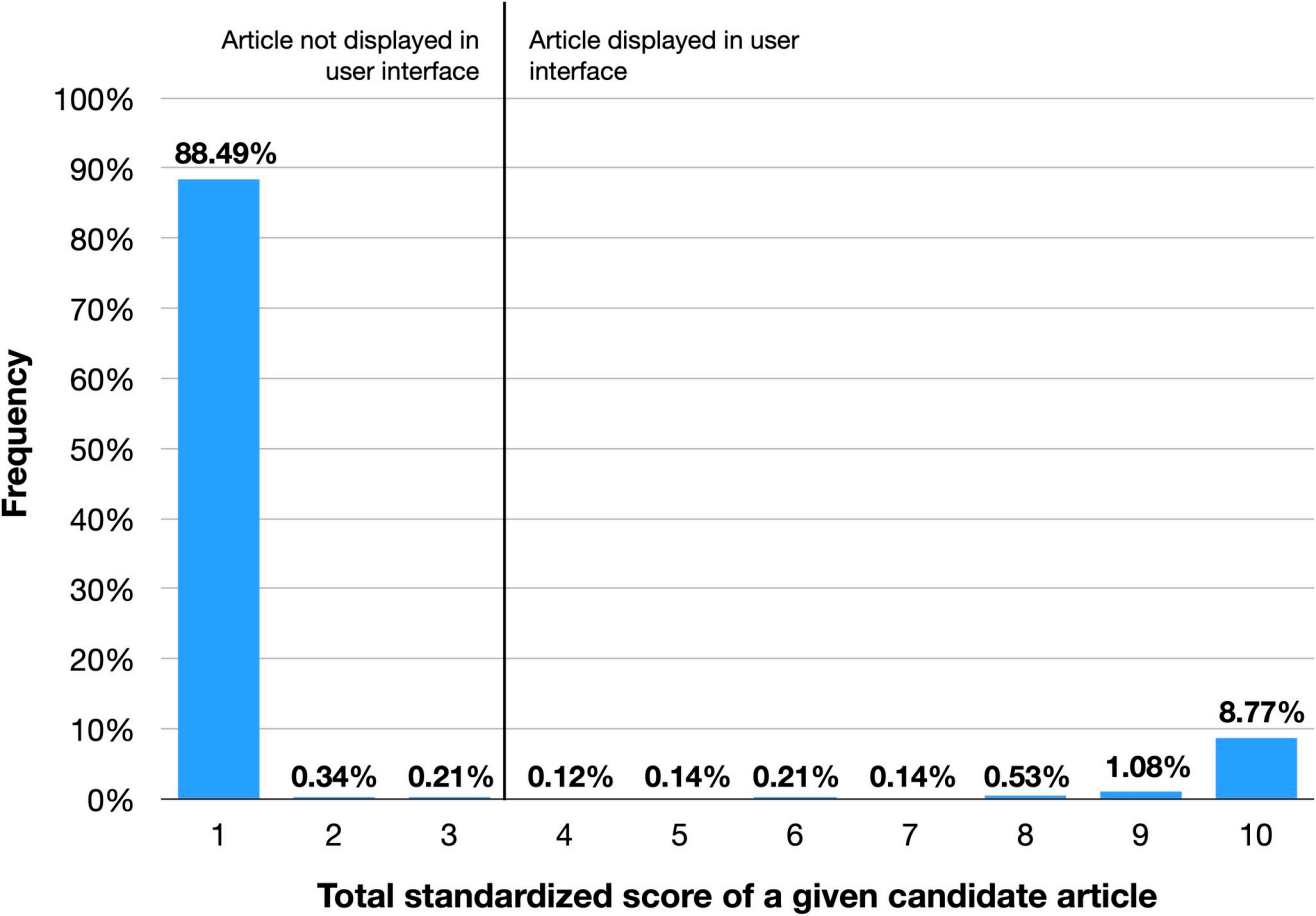
Frequency of total standardized article scores. Candidate articles with scores of 3 or below are generally not displayed in the user interface to the end user. This threshold is controlled in the application.properties file. The score displayed here is for total standardized score as opposed to total article score without clustering, which is analyzed in **Fig 11**.

Over 88% of candidate articles had a total-article-score-standardized of the lowest value of 1, meaning these articles are highly unlikely to be written by our scholar of interest. Almost 69.4% had a standardized score of 10, suggesting it is highly likely they were written by the scholar. See **Fig 10**.

**Fig 11:**
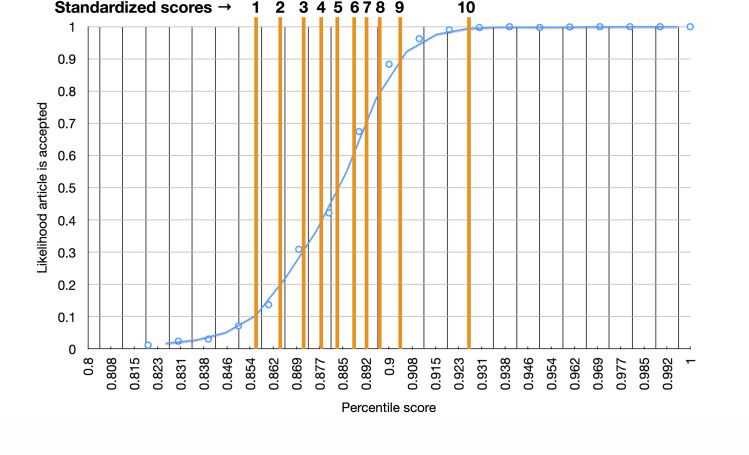
Optimization of standardized score thresholds. We have mapped all non-standardized scores of candidate article to a percentile. Each percentile corresponds to the percent likelihood an article with that score will be accepted by the user. Each 10% incremental increase in the likelihood of a percentile score increases the standardized score by 1. The likelihood an article will be accepted increases from 10% to 90% (y-axis) when the percentile score grows from 0.85 to 0.9.

Another goal of our analyses was to identify a set of intervals we could use for mapping a non-standardized score to a score on a scale between 1 and 10. As described in 2.5, we wanted to display a standardized score that corresponds to a percent likelihood that an article will be accepted. In **Fig 11**, we map percentile of non-standardized score (x-axis) against the likelihood the article was accepted (y-axis). This allows us to conclude that a percentile score of, say, 0.892 corresponds to a 70% likelihood that our scholar authored a publication. Using this logic, articles with this percentile score are assigned a standardized score of 7.

**Fig 12:**
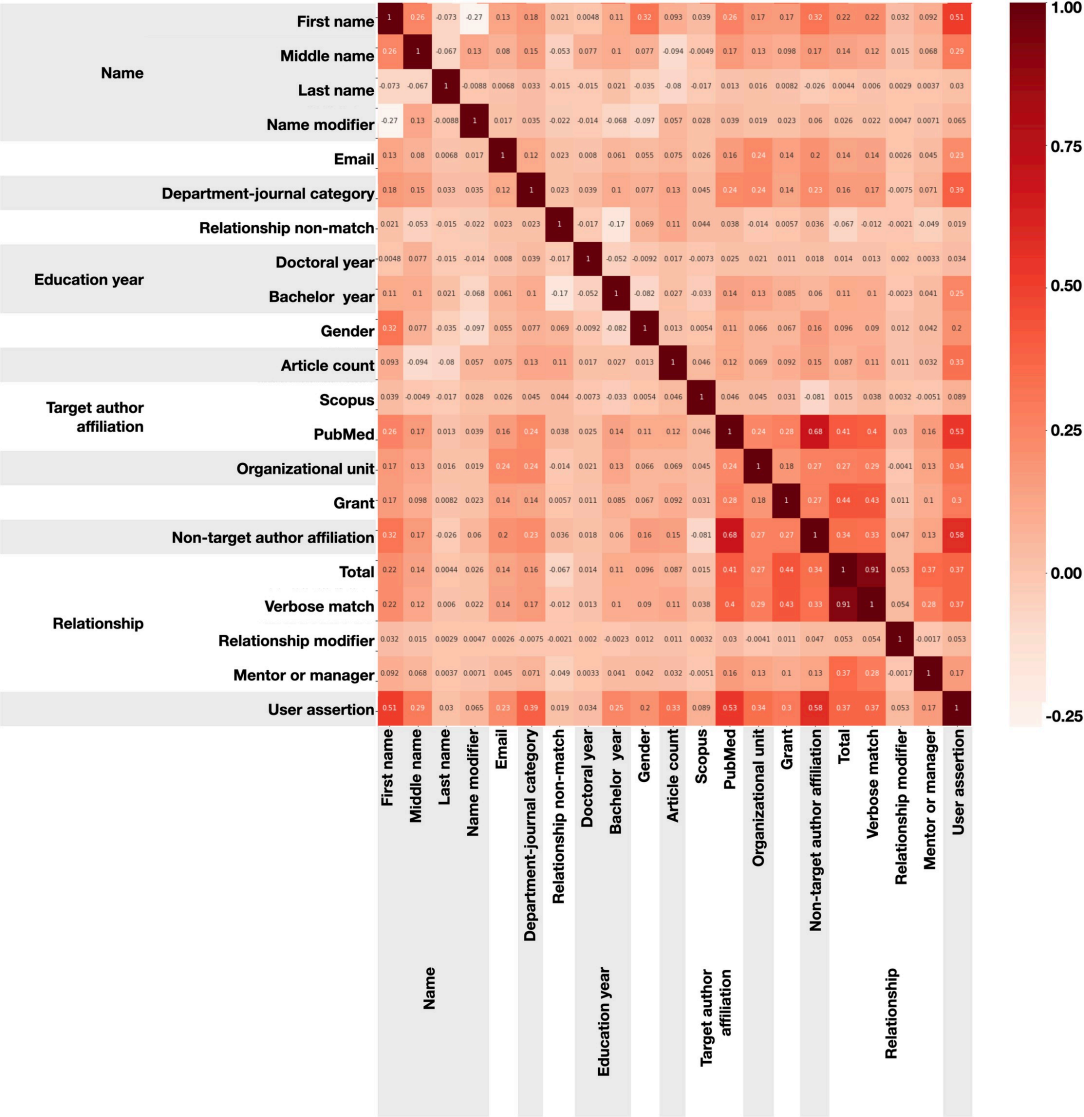
Correlation plot of features. This graph illustrates the extent to which any two attributes are correlated with each another, and with user assertion. Attributes that are correlated with the latter can be used to contribute to an article’s score. **S9 Appendix** contains descriptions of these values.

In **Fig 12**, we computed the correlations between different evidence subscores and user assertion. Cases where correlation is high are shaded in a dark red; low correlations are in a lighter color. For example, relationship-matching-score and relationship-verbose-matching-score have a correlation of 0.91. The value of this graph is that it shows, among other things, which attributes are independently associated with user assertion. Generally speaking, those that are highly and independently correlated with user assertion are most valuable to the overall performance of the algorithm. Conversely, if an attribute is highly correlated with another attribute, it does not offer much additional benefit.

**Fig 13:**
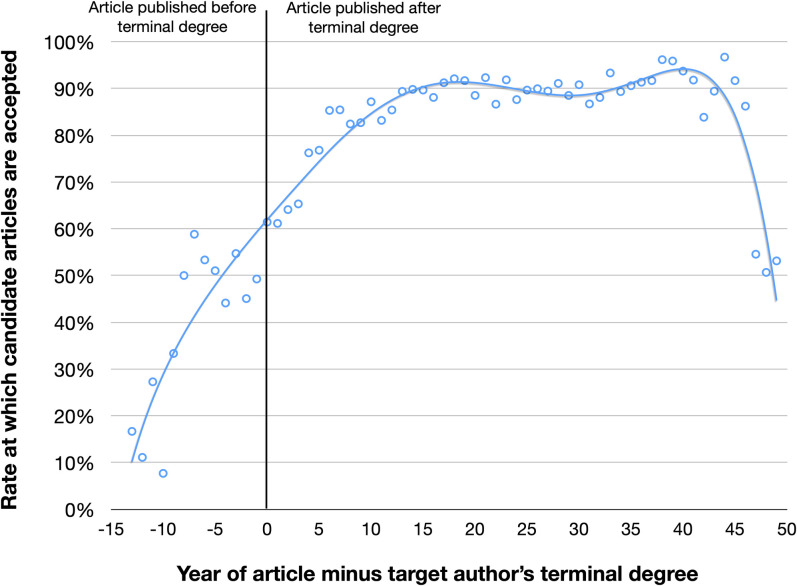
Association between a target author’s scholarly age and likelihood any given candidate article will be accepted. Candidate articles appearing 10 or more years after a scholar’s terminal degree are far more likely to have been written by the scholar of interest compared to those appearing within a year of receiving the degree.

One positive outcome of the SVM analysis was that we discovered there were a couple types of evidence subscores that were not meaningfully contributing to overall performance. An example of this is person-type-score. With our set of randomly selected scholars and their corresponding institutional identity data, person type evidence was determined to be largely redundant and less nuanced than education-year-score. Nonetheless, person type evidence could be used by other institutions which may have different needs. In **Fig 13**, we display how the year of a publication relative to a person’s scholarly age is associated with the likelihood that a candidate article will be accepted. According to our analysis, the likelihood a candidate article is authored by a person peaks and then plateaus between 10 and 40 years after an individual receives a terminal degree.

## 5. Discussion

### 5.1. Key findings

With the latest version of ReCiter, we have successfully created a publication management system, which is capable of predicting the publications authored by a diverse group of scholars at Weill Cornell Medicine with approximately 98% accuracy. Our system can lessen the workload for both administrators and scholars, providing publication metadata that can be used across public-facing profiles, reports, biosketches, and MyNCBI profiles.

ReCiter is a highly accurate, open source publication management algorithm designed for academic institutions with limited need for custom development. It innovatively uses a range of institutionally-maintained identity data to suggest which publications in PubMed a scholar has authored. Each of its components are needed for adoption by institutions. Additionally, because the key components are available as individual services, one could swap out one for another.

ReCiter retrieves and scores candidate articles for a given scholar, including those with unexpected or missing affiliations, as well as cases where article metadata is missing or highly misleading. ReCiter effectively uses the weights of a support vector machine analysis to score the available evidence.

ReCiter uses a wealth of institutionally-maintained identity data to suggest and score articles; provides an evidence-based probability for each of the candidate articles; offers a human-readable explanation for why a given article was suggested; and, reliably identifies a target author’s rank or position.

### 5.2. Weaknesses

While a significant subset of records in the Gold Standard had been validated by administrators and the scholars themselves including during the College’s annual review cycle, this analysis relies on a gold standard reviewed and updated by third parties. Ideally, all of the publication assertions would be validated by the scholars themselves. Scholars’ reluctance to curate their publications in a consistent and timely manner is ironically the very driver for the creation of this software.

ReCiter uses a variety of institutionally-maintained identity information to score its suggestions. We have attempted to construct a data model generic enough to accommodate the range of identity data maintained by a diverse array of institutions. For example, the relationships attribute could be populated with any variety of cases where a known association is likely to appear as a co-author. Yet, the most valid test of the durability of this model can be proven when this algorithm is used more widely.

One potential shortcoming is that many institutions may not have access to much of the identity data available in its identity data model. As a result, the accuracy of their system’s suggestions could suffer. One idea we contemplated is programmatically identifying and proposing candidate features such as email addresses, ORCID identifiers, name aliases, names of co-authors, etc. and then soliciting feedback from users. As Weill Cornell Medicine assists our Clinical Translational and Science Center in identifying publications for thousands of individuals about whom we have little data, this measure may significantly help.

Another challenge relates to what a fair method should be for computing precision for individuals with very common names. Yi Wang is a faculty at Weill Cornell. As of July 2020, a PubMed search for Wang Y[au] returns approximately 157,000 candidate records, with 3.7% of PubMed articles [33] having a “Y. Wang” as an author. Suppose a Yi Wang has authored only two papers, and we correctly identify those two and erroneously recommend 40 additional publications for this scholar. If ReCiter made a practice of counting those 157,030 records as true negatives, the recall would be 100%, the precision almost 5%, and the overall accuracy would be over 50%. For many, that figure may seem misleading. There may be no ideal approach for reporting ReCiter’s precision for common names. For now, ReCiter only counts publications from articles that are actually retrieved and scored–unless they are false negatives. Such articles are factored into the overall accuracy score even if they are not retrieved.

We note that a subset of publications that we judged to be correctly identified by ReCiter were not recorded in the legacy reporting database. However, this was relatively uncommon, as we discuss in the Methods, occurring for 20.2% of papers that were ultimately accepted. Less than 1% of papers were asserted to be authored by a scholar in our legacy database, but upon review by a librarian, were judged not to be. Ideally, the authority for which scholars wrote which papers would be curated entirely outside of the system being evaluated. We considered excluding all such papers from the analysis, but, we decided against that. We reasoned that we would never have access to both rich identity data and perfectly assigned publication metadata for the more than 16,000 papers authored by 500 scholars.

We recognize that ReCiter could more effectively use machine learning technologies when it comes to institutional disambiguation, assignment of publication type, and citation deduplication (i.e., Article A in PubMed refers to the same object as Article B in Scopus), among others. In particular though, we believe that ReCiter’s accuracy could be greatly improved by using a machine learning approach for clustering. At present, ReCiter does not use keywords from article title, journal title, or abstract. This absence represents a significant opportunity for improvement, especially for clustering.

### 5.3 Future work

Going forward, our team intends to deploy integrations with additional bibliographic sources. These integrations will also insulate developers from the complexity and the quirks of unwieldy source systems, and return data in a predictable, easy-to-parse format.

We have heard from a number of parties who are interested in generating suggested lists of scholarly articles from bibliographic sources that offer coverage of the social sciences and humanities. At present, ReCiter is optimized for PubMed, so this would require some dedicated effort. A source that had more complete *cites* and *cited by* data would be particularly attractive as ReCiter only uses *cited by* data recorded in PubMed Central, which contains less than 20% of PubMed.

For this analysis, we did not analyze how accepted and rejected publications affect the overall score of other candidate articles. These data are used in the average-clustering-score, described above, but they did not figure in our analyses. Anecdotally, we have noticed this feedback definitely improves the accuracy of scores of articles that were not accepted or rejected. The net effect is that fewer candidate articles have ambiguous authorship.

We realize that the validity of the approach we describe here would be more compelling if we could demonstrate that ReCiter can produce accurate lists of publications at other academic institutions, and show that implementing ReCiter saves administrators’ and scholars’ time, and results in more accurate outcomes. We intend to study these questions in future efforts.

## Supporting information

S1 Appendix Sample output file using Feature Generator API. This file represents the output of ReCiter’s Feature Generator API. With Feature Generator, both article and evidence metadata are included. Note that we set the total-article-score-standardized was set to the minimum of 1. This means all articles were included in the output. If this value was higher, the calculated precision would be higher as well.
(JSON)

S2 Appendix Weights used by ReCiter following SVM-based weight optimization. (CSV)

S3 Appendix Candidate record count for common names. 86 records had name lookups that exceeded the threshold for a "lenient" mode lookup and were looked up using "strict" mode.
(TXT)

S4 Appendix Bash script for downloading records using Feature Generator API. This script allows users to output the result of multiple calls to the Feature Generator API.
(SH) Click here for additional data file.

S5 Appendix ML_Model_Test_upload.py. A Python script which transforms JSON output from ReCiter’s Feature Generator API into a set of flat CSV files.
(PY) Click here for additional data file.

S6 Appendix ReCiterAnalysis.sql. Database model for storing output of Feature Generator API. Includes some sample data.
(SQL)

S7 Appendix Jupyter Lab notebook. Notebook accepts CSV files as inputs and, given user assertions, can be used to compute optimized weights for scores.
(IPYNB)

S8 Appendix Application.properties file with SVM-optimized weights. (TXT)

S9 Appendix Descriptions of scores used in Fig 12. (CSV)
